# Effect of oat bran on time to exhaustion, glycogen content and serum cytokine profile following exhaustive exercise

**DOI:** 10.1186/1550-2783-7-32

**Published:** 2010-10-18

**Authors:** Felipe F Donatto, Jonato Prestes, Anelena B Frollini, Adrianne C Palanch, Rozangela Verlengia, Claudia Regina Cavaglieri

**Affiliations:** 1 Health Science Faculty, Methodist University of Piracicaba, São Paulo, Brazil; 2 Graduation Program in Physical Education - Catholic University of Brasilia, - Brasília/DF/Brazil; 3 Molecular Biology of the Cell Group, Institute of Biomedical Sciences, Department of Cell and Developmental Biology, University of São Paulo, Brazil

## Abstract

The aim of this study was to evaluate the effect of oat bran supplementation on time to exhaustion, glycogen stores and cytokines in rats submitted to training. The animals were divided into 3 groups: sedentary control group (C), an exercise group that received a control chow (EX) and an exercise group that received a chow supplemented with oat bran (EX-O). Exercised groups were submitted to an eight weeks swimming training protocol. In the last training session, the animals performed exercise to exhaustion, (e.g. incapable to continue the exercise). After the euthanasia of the animals, blood, muscle and hepatic tissue were collected. Plasma cytokines and corticosterone were evaluated. Glycogen concentrations was measured in the soleus and gastrocnemius muscles, and liver. Glycogen synthetase-α gene expression was evaluated in the soleus muscle. Statistical analysis was performed using a factorial ANOVA. Time to exhaustion of the EX-O group was 20% higher (515 ± 3 minutes) when compared with EX group (425 ± 3 minutes) (p = 0.034). For hepatic glycogen, the EX-O group had a 67% higher concentrations when compared with EX (p = 0.022). In the soleus muscle, EX-O group presented a 59.4% higher glycogen concentrations when compared with EX group (p = 0.021). TNF-α was decreased, IL-6, IL-10 and corticosterone increased after exercise, and EX-O presented lower levels of IL-6, IL-10 and corticosterone levels in comparison with EX group. It was concluded that the chow rich in oat bran increase muscle and hepatic glycogen concentrations. The higher glycogen storage may improve endurance performance during training and competitions, and a lower post-exercise inflammatory response can accelerate recovery.

## Background

The importance of dietary carbohydrates (CHO) in sporting performance was shown in the classical gaseous exchange experiments and biopsy studies, in which increasing exercise intensity utilises a greater proportion of CHO [1,2]. These data provided a major breakthrough for the science of sports nutrition, as it enabled the exact amount of CHO for athletes to be quantified.

The recommendations concerning carbohydrates (CHO) for athletes are around 6 g-10 g/Kg/day [3-5] and these quantities vary in accordance with the quantity of body mass, gender, volume and intensity of the training. According to Tarnopolsky [3] elite athletes train around 8 to 40 hours per week, exponentially increasing their nutritional needs. The International Olympic Committee (IOC) recommends that: "following a diet rich in carbohydrates days before a competition can help to increase sporting performance, particularly when the exercise is kept up for longer than 60 minutes" [6].

Exhaustive endurance exercise can induce immune disturbances and consequently increase susceptibility to upper respiratory tract infections [7]. Several mechanisms have been proposed in an attempt to explain the susceptibility of athletes to respiratory infections. Cortisol contributes only minimally to the exercise induced rise in liver glucose output [8], while it plays a role in immune disturbances [9,10]. Several components of the innate immune system are compromised during single or repeated sessions of exercise stress.

Physical exercise can affect the levels of systemic cytokines, such as TNF-α [11-13], interleukin 1 beta (IL-1β) [12], IL-6 [12-16], interferon and others [11]. Recently, it has been suggested that the disruptions in the balance between pro- and antiinflammatory cytokines may lead to a loss of inflammatory control, with possible implications for overall immune system function [17,18]. The effect of ingesting carbohydrates during long duration exercises, with the purpose of attenuating immune suppression is well established [6,12-14].

Cereals oat bran has a high nutritional quality, an naturally source of CHO [19], rich in proteins, unsaturated fatty acids, vitamins, and complex starches that comprise the part with the largest quantity of soluble fiber. Another important nutrient in oat bran is β-Glucan, and has well-documented stimulation effects on the immune system. Also may help enhance immune resistance to various viral, bacterial, protozoan, and fungal diseases [20]. Animal studies show that oat β-glucan can offset exercise-induced immune suppression and decrease susceptibility to infection during heavy training [21]. Therefore, the aim of this study was to evaluate the effect of oat bran supplementation on time to exhaustion, glycogen stores and cytokines profile in rats submitted to training.

## Materials and methods

### Experimental groups

All experiments were conducted according to the policy of the American College of Sports Medicine on Research with Experimental Animals. Two-month-old male *Wistar *rats (*Rattus novergicus var. *albinus, Rodentia, Mammalia) with a mean ± SEM weight of 200 ± 5 g were used. The animals had free access to water and were fed a commercial chow for rodents (NUVILAB, Purina^®^) *ad libitum*. The animals were kept in collective cages (3 rats per cage) at a constant temperature of 23 ± 2°C, and a cycle of 12 hours light/12 hours darkness, with light from 06:00 h to 18:00 h (in pathogen-free housing). Before the experimental period began, the animals underwent 48 hours of adaptation to the research laboratory conditions. The 27 animals were divided into 3 groups (n = 9 each group): sedentary control group that underwent no physical training (C), an exhaustion group that received a control chow (EX) and an exhaustion group that received a chow supplemented with 30% of soluble oat bran fibers (EX-O). All experiments conducted on animals were previously approved by the Ethics Committee on Animal Testing, Federal University of San Carlos.

### Chow Preparation

For eight weeks, the animals received chow prepared weekly, stored and analyzed. Only carbohydrate, protein, lipid and fibre content in chow were analyzed. Every care was taken to ensure that these diets remained homogeneous during the entire experimental period. The chow was prepared from a commercial chow (NUVILAB, Purina^®^) which, after milling, had its fibre content adjusted by adding 30% of oat bran (Oat bran Quaker^®^), or 300 g/Kg of standard commercial chow. The chow was characterized according to the procedures of Cavaglieri [22]. Table 1 demonstrates the chow compositions.

**Table 1:** Nutritional Composition in grams (g) of the chows used.

NUTRIENT	CONTROL	%	EXPERIMENTAL	%
**Protein (g)**	18	24.8	17.4	23.5
**Fat(g)**	4	12.4	4.9	14.8
**Carbohydrate(g)**	45.5	62.7	45.6	61.6
**Total fibers (g)**	21.9	-	18.9	-
**Insoluble fibers (g)**	18	82	14.4	76.1
**Soluble fibers (g)**	3.9	17.8	4.5	23.8

### Exercise Protocol

The animals were submitted to a 5-day period of adaptation to the liquid environment (5 minutes on the first day, 15 minutes on the second, 30 minutes on the third, 45 minutes on the fourth and 60 minutes on the fifth), in accordance with Sampaio-Barros [23]. Importantly, the control groups were submitted in contact with water, but did not perform the exercise. This was done to equalize the stress compared to the exercised group. A tank was used to perform the swimming sessions, were made of plastic and did not have places where animals could cling to. This was necessary to achieve constant exercise. The water temperature was monitored at approximately 28 ± 2°C. After adaptation, the training consisted of 60 minutes of daily swimming, five days per week, for eight weeks, performed in the afternoon between 14:00-17:00. The moderate intensity they used a load of 5% of their body weight strapped to their backs, which corresponds to intensity below the point of inflection of the lactate threshold curve. At the end of eight weeks training, the animals were submitted to the exhaustion test, characterized by being incapable of keeping themselves on the surface of the water [24,25].

### Animal sacrifice and sample collection

Immediately after the exhaustion test, the animals were sacrificed by decapitation. During exsanguination, the mixed arteriovenous blood was collected in heparinized tube and chilled on ice. Blood was then spun at 500*g *for 15 min to obtain plasma for cytokine and corticosterone analyses. In the following order, the liver, soleus and white and red gastrocnemius muscle were collected and stored at -70°C until the time of measurement of hepatic and muscle glycogen. The white and red portion of the gastrocnemius was divided throughout the major colour of muscle fibres.

### Determination of muscle and hepatic glycogen concentrations

The muscle samples were digested in 30% KOH at 100° C and glycogen was precipitated by passage through ethanol. Between each precipitation the sample was centrifuged at 3000 rpm for 15 minutes. The precipitated glycogen was submitted to acid hydrolysis in the presence of phenol. The values were expressed in mg/100 mg of wet weight, using the Siu method [26].

### Determination of serum cytokines

After the period of supplementation and training, measurements of IL-6, TNF-α and IL-10 in plasma were made by ELISA using the R & D Systems Quantikine High Sensitivity kit (R&D Systems, Minneapolis, MN, USA) for each cytokine. The intra-assay coefficient of variance (CV) was 4.1 - 10%, the inter-assay CV was 6.6 - 8%, and the sensitivity was 0.0083 pg/ml [13]. The duplicate plasma aliquots for all cytokines analysis were used.

### Corticosterone determination

Plasma corticosterone was determined by ELISA, using the Stressgen kit (Corticosterone ELISA KIT Stressgen@), Michigan, USA). The sensitivity range of the assay was 32-20.000 ng/ml. The duplicate plasma aliquots for hormone analysis were used.

### Determination of glycogen synthetase-alpha (GS-α) mRNA expression in the soleus muscle

#### Total RNA extraction

Total RNA was obtained from 100 mg of soleus muscle. The tissue were stored at -70°C until the time of measurement. Cells were lysed using 1 mL of Trizol reagent (Life Technologies, Rockville, MD, USA). After incubation of 5 min at room temperature, 200 μL chloroform was added to the tubes and centrifuged at 12,000 × g. The aqueous phase was transferred to another tube and the RNA was pelleted by centrifugation (12,000 × g) with cold ethanol and air-dried. After this, RNA pellets were diluted in RNase-free water and treated with DNase I. RNAs were stored at -70°C until the time of measurement. RNA was quantified by measuring absorbance at 260 nm. The purity of the RNAs was assessed by the 260/280 nm ratios and on a 1% agarose gel stained with ethidium bromide at 5 μg per mL [27].

### RT-PCR

RT-PCR was performed using parameters described by Innis and Gelfand [28]. The number of cycles used was selected to allow quantitative comparison of the samples in a linear manner. For semi-quantitative PCR analysis, the housekeeping β-actin gene was used as reference. The primer sequences and their respective PCR fragment lengths are: GSK3-α sense: AATCTCGGACACCACCTGAGG - 3'; anti-sense: 5'GGAGGGATGAGAATGGCTTG - 3'. Control: β-actina sense: 5'-ATGAAGATCCTGACCGA GCGTG-3'; anti-sense: 5'- TTGCTGATCCACATCTGCTGG-3'. Published guidelines were followed to guard against bacterial and nucleic acid contamination [29].

### Analysis of the PCR products

The PCR amplification products were analyzed in 1.5% gels containing 0.5 μg per mL of ethidium bromide and were electrophoresed for 1 h at 100 V. The gels were photographed using a DC120 Zoom Digital Camera System from Kodak (Life Technologies, Inc., Rockville, MD, USA). The images were processed and analyzed in the software Kodak Digital Science 1D Image Analysis (Life Technologies). PCR band intensities were expressed as Optic Density (OD) normalized for β-actin expression. Data are presented as a ratio compared with the respective controls, which received an arbitrary value of 1 in each experiment.

### Statistical analysis

Data are presented as mean ± SEM (standard error of the mean). The normality of distribution of all parameters was checked with the Kolmogorov-Smirnov test and by the homocedasticity test (Bartlett criterion). All variables presented normal distribution and homocedasticity, thus the two-way ANOVA test was used, (taking into consideration the variables exercise × oat bran enriched diet) and when the difference presented was significant, Tukey's *post hoc *test was used. A significance level of *p *≤0.05 was used for all comparisons. The software package used was SPSS for Windows version 10.0.

## Results

### Time to Exhaustion

**Figure 1:**
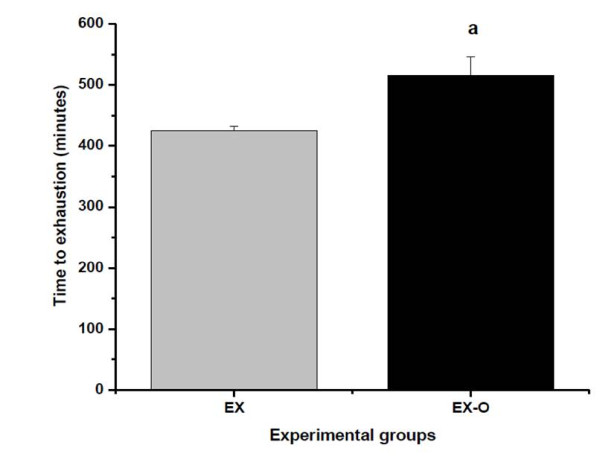
**Time to exhaution on experimental groups**. a = statistical difference to exhaution group (EX)

The time to exhaustion of the EX-O group was 515 ± 30 minutes and 425 ± 30 for the EX group (p = 0.034). This represented a 20% higher exhaustion time for the EX-O group when compared with the EX group. Figure 1

### Corticosterone and Cytokine Concentrations

**Figure 2:**
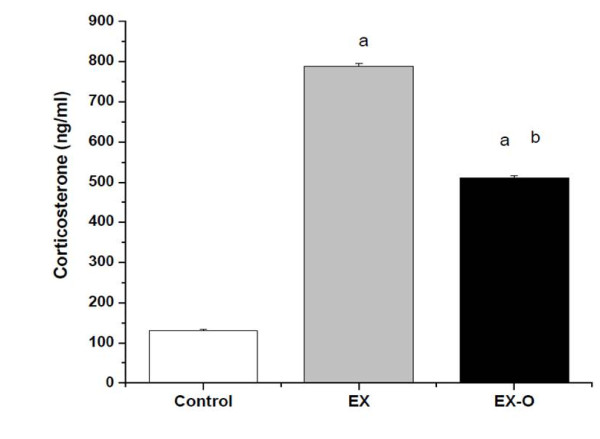
**Corticosterone levels in experimental groups**. a = statistical difference to control group b = statistical difference to EX group

Corticosterone levels were significantly elevated after exercise to exhaustion compared with the control group. The EX group presented significantly higher corticosterone levels compared with the EX-O group, (p = 0.039) (figure 2). Similarly, after exercise IL-6 was increased in EX and EX-O compared with the control. The EX-O group showed lower levels of IL-6 compared with the EX group, (p = 0.001) (Table 2). The serum levels of TNF-α were significantly decreased after exercise in the EX and EX-O groups compared with the control group. However, no statistically significant differences were observed between EX and EX-O for TNF-α serum levels (Table 2). IL-10 serum levels were increased after exercise compared with the control group, and EX presented significantly higher levels of IL-10 as compared with EX-O (p = 0.032) (Table 2).

**Table 2:** Plasma cytokine concentration in experimental groups.

(pg/ml)	C	EX	EX-O
**IL-6**	11.2 ± 17	163 ± 2.7*	127 ± 3.6*^#^
**IL-10**	50.5 ± 9.4	328.5 ± 78*	84.3 ± 53.4*^#^
**TNF-a**	31.1 ± 1.34	5.58 ± 1.0*	2.6 ± 0.4*

Values are presented as mean ± standard error of the mean. Control (C), exhaustion (EX) and exhaustion treated with oat bran (EXO) groups, (n = 9), p ≤ 0.05. IL-6 = interleukin-6; IL-10 = interleukin-10; TNF-a = Tumor necrosis factor-a. *Statistically significant difference compared with C group; ^#^statistically significant difference compared with EX group.

### Glycogen Concentrations and expression of Glycogen synthetase mRNA

With regard to hepatic glycogen, the exhaustion test diminished the hepatic glycogen of the EX-O group by 61% and that of the EX group by 87% in comparison with the control group. In the comparison between the exercise groups, EX-O presented a 67% higher hepatic glycogen concentrations when compared with EX (p = 0.022), as shown in Table 3.

**Table 3:** Hepatic and muscle glycogen concentration (mg/100 mg)

	C	EX	EX-O
**Hepatic glycogen**	5.5 ± 1.06	0.8 ± 0.09*	2.9 ± 0.64*^#^
**White gastrocnemius**	0.61 ± 0.06	0.12 ± 0.01*	0.14 ± 0.03*
**Red gastrocnemius**	0.53 ± 0.05	0.14 ± 0.02*	0.16 ± 0.04*
**Soleus**	0.70 ± 0.05	0.15 ± 0.06*	0,37 ± 0.04*^#^

Values are presented as mean ± standard error of the mean. Control (C), exhaustion (EX) and exhaustion treated with oat bran (EX-O) groups, (n = 9), p ≤ 0.05. *Statistically significant difference compared with C group; ^#^statistically significant difference compared with EX group.

**Figure 3:**
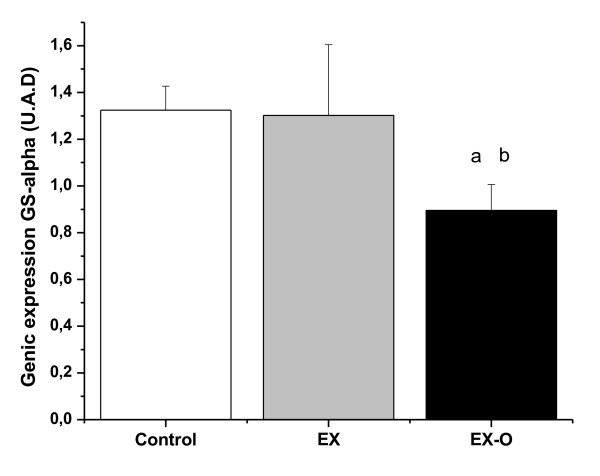
**Glucogen synthetase gene expression**. a = statistical difference with control group b = statistical difference with EX group

There was a decrease of 47% in soleus muscle glycogen concentrations for the EXO group (p = 0.043), and of 78.5% for the EX group (p = 0.036) when compared with the control group. When comparing the exercise groups, EX-O presented a 59.4% higher soleus glycogen concentrations than the EX group (p = 0.021, see Table 3). Gene expression of GS-alpha (U.A.D) in the C group was 1.32 ± 0.1, EX group 1.30 ± 0.3 and EXO group 0.89 ± 0.1 (Figure 3). Furthermore, the EX-O group presented lower levels of glycogen synthetase-α enzyme in the soleus muscle when compared with the EX group (p = 0.013).

The quantity of glycogen in the white gastrocnemius muscle decreased by 77% in the EX-O (p = 0.011), and 80% in the EX group (p = 0.037) when compared with the control. There were no significant differences between EX-O and EX in the glycogen concentrations of the white gastrocnemius muscle (Table 3).

The exhaustion test diminished the muscle glycogen concentrations of the red gastrocnemius by 69.8% in the EX-O group, and by 73.5% in the EX group (p < 0.05), when compared with the control group. In the comparison between the exercise groups, no significant differences were observed (Table 3).

## Discussion

The aim of this study was to evaluate the effect of oat bran supplementation on time to exhaustion, glycogen stores and cytokines profile in rats submitted to training. The animals did not receive any type of carbohydrate during the time they were performing the exercise, only *ad libitum *food during the eight weeks of training. In the present investigation, the exercise protocol used was one hour of daily swimming, 5 days per week during two months At the end of the eight weeks, were performed the test exhaust. For the impact in performance, the carbohydrate content should be equal, there by the experimental chows had the same quantity of carbohydrates, being 45.5 g/100 g for the control and 45.6 g/100 g in the experimental chow.

Similarly, in the chows of the present study, one can note a lower quantity of total fibres in the experimental chow (18.9 g) and a higher quantity in the soluble portion (4.5 g). Although the total fibre content was higher (21.9 g) in the control diet, the quantity in the soluble part was lower (3.9 g).The difference in available carbohydrate (avCHO = total carbohydrate minus fiber) is the better explanation: control chow has 45.5 cho-21.9 fiber = 23.6 g avCHO while the oat bran diet contains 45.6 cho-18.9 fiber = 26.7 g avCHO. It is a 13% increase in the oat bran chow.

Changes in the intestinal microflora that occur with the consumption of prebiotic fibres may potentially mediate immune changes via: the direct contact of lactic acid bacteria or bacterial products (cell wall or cytoplasmic components) with immune cells in the intestine; the production of short-chain fatty acids from fibre fermentation; or by changes in mucin production. The link between oat bran and immune system its regard with the content of β-glucan, especially water-soluble β-glucan. This soluble fiber can enhance the activities of both the innate and specific immune system components via direct activation of specific receptors on macrophage, neutrophils, and NK cells [30,31] or indirectly after activation of pinocytic M-cells located in the Peyer's patches of the small intestine [32,33]. There is increasing evidence that fermentable dietary fibres and the newly described prebiotics can modulate various properties of the immune system, including those of the gut-associated lymphoid tissues (GALT).

In published data on the immune system of the same experimental group, Donatto [34] demonstrated that the EX-O group presented better phagocytic capacity of peritoneal macrophages, increased amount of lymphocytes from lymph nodes and shows less leukocytosis after exhausting exercise. We found no side effects in this study, including no increase in the plasma concentration of pro inflammatory cytokine. β-glucan found in oat bran could not exaggerate the inflammatory response to severe exercise.

Glycogen metabolism is largely controlled by the actions of glycogen synthase and glycogen phosphorylase enzymes [35]. The gene expression of Glycogen synthase increased after both resistance and aerobic training, but not when aerobic exercise was combined with a high CHO diet in comparison with diet without exercise [36]. In the present study, we found a lower expression of the glycogen synthetase enzyme in the soleus muscle in the EXO group. Probably, the higher glycogen levels in the soleus muscle had an important relationship with the impaired glycogen synthetase expression. It may reflect a lower need for re-synthesis [37] since this group presented higher glycogen concentrations in the soleus when compared with exhaustion of the non-oat bran enriched diet group (EX).

The oat bran is a nutritional search of dietary fiber, especially soluble fiber and this nutriente may retard the absorption of nutrients by the intestinal villosities [38]. In this case, the glucose absorption metabolism had a modulation to lower and constant delivery to blood circulation and this could be responsible for a more efficient replacement of muscular glycogen during a longer recovery period [37,39,40]. There was a correlation between the low levels of glycogen and higer corticosterone and IL-6. During prolonged and exhausting physical exercises (duration in excess of 90 minutes), the IL-6 has a close relationship with the amount of muscle glycogen and regulation of the homeostasis of blood glucose during long duration exercises. Muscular glycogen and blood glucose are the major sources of substrates for oxidative metabolism, and the immune depletion and fatigue coincides with their depletion, due to the low availability to the skeletal muscle and the central nervous system [41-45].

In the EX group glycogen levels were low while IL-6 and corticosterone were high. In contrast, the inverse was observed in the EX-O group which had higher levels of muscle glycogen and lower levels of corticosterone and IL-6. These results were shown in EX group, since the animals swam an average of 11 hours, ending in a worst metabolic condition On the other hand, EX-O swam an average of 2 hours longer, totalling 13 hours of physical exercise with lower levels of IL-6 and corticosterone, consequently at the end of exercise protocol shows an better condition.

Plasma concentration shows the total secreted of some products like corticosterone and cytokines by all tissues, but does not know the source of secretion. Unfortunately, some of the shortcomings of this study were not to analyze the cytokines levels in different tissues. One of the hypotheses regarding the mechanism of central fatigue is that IL-6 can exert direct influence on hypothalamus-pituitary-adrenal axis, thereby increasing ACTH-cortisol release [15,46]. Moreover, the different kits used to measure IL-6 plasma levels difficult the comparison between studies.

The exercise protocol used in the present study modulated the serum levels of TNF-α, as a result of the lower levels of TNF-α in the trained groups when compared with the control group. In 1999, Ostrowski and colleagues [47] presented the plasma cytokines profile after a marathon race (mean duration 3: 26 (h: mi.), with increased levels of TNF-a, IL-6 and IL-10. Their study revealed a proinflammatory and anti-inflammatory profile after a marathon race. Pedersen [16] suggested that regular exercise modulates some pro-and anti-inflammatory cytokines, induces suppression of TNF-alpha and thereby offers protection against exacerbated inflammation.

Unfortunately, the levels of cytokines in the adipose tissue and muscle were not measured, so that the source of cytokine production cannot be determined. This is an important issue because there is a different production of cytokines in muscle and adipose tissue, and exercise has an influence in this process. Rosa Neto et al. [48] showed an anti-inflammatory effect of strenuous exercise on muscle and a pro-inflammatory effect on adipose tissue.

In this sense, Pedersen (16) revealed an anti-inflammatory effect of acute physical exercise, characterized by an increased circulating level of IL-10, IL-1 receptor antagonist (IL-1ra) and soluble receptor of TNF (TNFRs). Lira et al. [49] showed an anti-inflammatory profile on adipose tissue in rats submitted to aerobic training (decreased TNF-alpha and increased IL-10 levels). In the present study, the combination of exercise with oat bran induced a decrease on TNF-alpha levels associated with an increase in IL-10 serum levels (anti-inflammatory cytokine).

These results show that oat bran, how another search of carbohydrate can directly influence the metabolic stress induced by exhaustive long duration exercise, saving the energy reserves and promoting better performance during exercise, thus corroborating findings in the literature [7,15,42,44]. If our data can be clinically translated, they may lead to an important new nutritional strategy to boost the immune system and decrease the risk of infection that can be a problem in athletes and military personnel who are often exposed to combinations of severe physical, psychological, and environmental stressors. In practical terms, athletes who practice long duration exercises may maintain the stocks of glycogen at more favourable concentrations to perform daily training sessions, by means of ingesting carbohydrate, vitamins, minerals, and β-glucan in the form of oat bran.

## Conclusions

In summary, it could be concluded that soluble fibres (i.e. chow rich in oat bran) increased muscular and hepatic glycogen concentrations, and this resulted in a longer time to exhaustion with an associated reduction in pro-inflammatory cytokines. In practical terms, these results demonstrate the importance, not only of the quantity of carbohydrates, but also the balance of dietary fibre content. Further studies conducted in athletes and animal models, using oat bran supplementation are necessary, with the aim of assessing improved performance, in view of the possible positive effects found in the present research.

## Competing interests

The authors declare that they have no competing interests.

## Authors' contributions

CC: dissertation guidance, interpretation of the data and preparation of experimental chow; JP: randomization of the protocol training of animals, literature review and ELISA assays assistance; FA and DR: animal training assistance; RV and AP: molecular biology assays. All authors read and approved the final manuscript.
